# Bioactive extracts and association with C and N in *Eleutherococcus senticosus* subjected to chitosan nanoparticles in contrasting light spectra

**DOI:** 10.1371/journal.pone.0277233

**Published:** 2022-12-01

**Authors:** Shenglei Guo, Hexiang Wang, Yawen Sui, Xiubo Liu, Long Tan

**Affiliations:** 1 College of Pharmacy, Heilongjiang University of Chinese Medicine, Harbin, China; 2 Key Laboratory of Basic and Application Research of Beiyao (Heilongjiang University of Chinese Medicine), Ministry of Education, Harbin, China; 3 College of Jiamusi, Heilongjiang University of Chinese Medicine, Jiamusi, China; VIT University, INDIA

## Abstract

Bioactive compounds are major reasons for the value of *Eleutherococcus senticosus*, which can be modified by different lighting spectra. Light-emitting diode (LED) provides lights with specific spectra which can interact with other treatments to impact plant bioactive production. Chitosan nanoparticle (CN) is a biopolymer derived from marine creatures. It’s usage may be a practical approach to cope with uncertainties in secondary metabolites induced by illumination. Carbon (C) and nitrogen (N) cyclings link plant eco-physiological performance and bioactive substance; hence their associations may reveal the mechanism of joint light-CN interaction. In this study, *E*. *senticosus* seedlings were raised under artificial lighting spectra from high-pressure sodium (HPS) lamps (44% red, 55% green, 1% blue) and white (44% red, 47% green, 8% blue) and red colored (73% red, 13% green, 14% blue) LED panels. Half of the seedlings received CN and the other half received distilled water as the control. Compared to the HPS spectrum, the red-light induced stronger shoot growth with greater biomass accumulation and higher water uptake but resulted in lower N concentration and biomass ratio in the root. The white light caused more biomass allocated to the root and strengthened stem C concentration. Stem eleutheroside B increased with shoot growth, while root eleutheroside B had a positive association with leaf C and stem protocatechuic acid had a negative association with leaf N. Having the CN treatment in white and red LED lights is recommended for increasing accumulation of bioactive compounds in the shoots and roots of *E*. *senticosus* seedlings, respectively.

## Introduction

Non-wood forest products (NWFPs) are natural products derived from forests rather than timber [1]. NWFP accounts for a significant proportion of household income in rural regions [2, 3]. Plants from the Araliaceae family are usually cultivated to create NWFPs due to their uses in food and medicine [4–10]. The *Eleutherococcus* genus encompasses 35 species in the natural habitats of China, Japan, Korea, and in the mild-climate zones of North America [11]. *Eleutherococcus senticosus* (Rupr. & Maxim.) Harms is an unique species from *Eleutherococcus* genus that is being authenticated by American Herbal Pharmacopoeia and European Pharmacopoeia (10.0 edition) [11, 12]. The root bark of *E*. *senticosus* was found to be a restorative tonic in Japan [13]. The thorny stem and branches were also found to be the source of extracts used for treating disease, tuberculosis, and bruises [14–16]. Natural *E*. *senticosus* habitats may have been impacted by climate, which induced bioactive extracts along a heterogenous spatial distribution pattern [9]. Therefore, carbon (C) and nitrogen (N) cycling in *E*. *senticosus* may differ by regional climate change. The plant factory is an alternative and stable environment to cultivate *E*. *senticosus* [17–20]. Technological details in plant factory have to be determined according to *E*. *senticosus*’ ecophysiological responses to controlled environment factors that benefit dry mass production and lower antioxidant activities.

Plants from the Araliaceae family are mostly shade tolerant in the understory layer with limited sunlight transmittance [6–8, 21, 22]. Low light is an inevitable factor in the habitat environment, which benefits accumulation of medicinal bioactive extracts, but restricts growth [6, 8, 23, 24]. In temperate forests, a low light intensity refers to photosynthetic photon flux density (PPFD) between 75–100 μmol m^-2^ s^-1^. [5, 6]. Therefore, low light irradiation is a fundamental aspect of an undergrowth and light quality in varied spectra plays a critical role. Therefore, the spectrum of low-intensity light is an important determinant of medicinal bioactive compounds in shade-tolerant plants. For example, both total flavonoid and saponin concentrations were found to be higher in *Alpinia oxyphylla* plants subjected to a red-light spectrum (30% red, 10%, green, 20% blue) [25]. *Allium victorialis* plants had higher concentrations of total flavonoids and saponins when subjected to a green-light spectrum (28% red, 71% green, and 1% blue) [26]. Saponin concentration were lower in the shoots of *Aralia elata* plants subjected to the green-light spectrum (26.6% red, 59.9% green, 13.5% blue) [8]. Regarding the highly varied responses of secondary metabolites in medicinal plants to different spectra, an additive may be needed to attain the desired amount of bioactive compounds.

Chitosan is a biological additive used in agricultural and horticultural plant factory systems. Chitosan is the product of alkaline deacetylation of chitin, which is derived from marine organisms such as insect cuticles, fungal cell walls, and crustacean shells [27]. Chitosan is the second most abundant polysaccharide next to cellulose [28]. Additives with chitosan oligosaccharide were reported to benefit plant growth and promote resistance against droughts [29], nitrogen remobilization [30], and increase nutrient uptake and utilization [31]. Chitosan based nanoparticles (CN) release bioactive molecules at a controlled speed, which was found to benefit plant growth and mitigate stress [28, 32]. The CN addition has been identified to promote protective secondary metabolites in response to biotic and abiotic stressors [33]. Therefore, CN can be used as an additive to potentially enhance secondary metabolite accumulation in cultivars in the plant factory environment. To our knowledge, one study tested the effect of chitosan addition on Araliaceae plants, i.e. *Aralia continentalis* Kitagawa [34]. Therein, root extracts intereacted with chitosan to increase antioxidant capacity. Hence, adding CN may promote the bioactive synthesis of secondary metabolites that have medicinal uses. More evidence is needed to verify the bioassay response of Araliaceae plants to CN addition. There is still uncertainty in the interaction of CN addition with light spectrum regarding contrasting outcomes with a joint [8] or a null effect [10].

*Eleutherococcus senticosus* (Eleuthero) (colloquially, ciwujia, Siberian ginseng, Devil’s shrub) is a small woody shrub in the Araliaceae family native to Northeast Asia. It has been used as a traditional herb medicine in China for over 2000 years and recently accounted for over 3% of the market share of the medicinal herb industry in North America [35]. *E*. *senticosus* contains many bioactive compounds that have medicinal uses. For example, they were reported to contain abundant amounts of bioactive metabolites such as phenylalanine (PHE) [36], protocatechuic acid [37], eleutherosides, chlorogenic acid, caffeic acid [38], isofraxidin [39], etc. These compounds can treat many illnesses due to their antioxidant abilities [40]; therefore, the accumulation of these bioactive compounds is one of the major goals when culturing *E*. *senticosus*. Although C and N metabolisms account for an important proportion of synthesis and accumulation of bioactive compounds in *E*. *senticosus*, little is known about the identifications of bioactive compounds and their association. In natural habitats, bioactive compounds in *E*. *senticosus* were found to show a significant plasticity that is shaped by understory environmental factors [9]. Specifically, some LED light spectra can modify the root growth of *E*. *senticosus* seedlings and the addition of chitosan can strengthen this effect [10]. The response of bioactive compounds to the interaction between some LED light spectra and chitosan is not clear. There still needs to be more research on the relationship between C and N metabolisms.

In this study, *E*. *senticosus* seedlings were cultured in a plant factory and tested for bioactive content and antioxidant activity. All seedlings were cultured using artificial illumination from sodium (HPS) and light-emitting diode (LED) lamps. HPS lamps have long been used to supplement illumination for woody plants to promote growth or enhance their quality [8, 19, 41, 42]. Alternatively, LED panels and lamps are being adapted by a growing number of plant managers for their lower energy consumption and higher efficacy in quality elevation [18, 25, 26, 43, 44]. Light spectra and CN addition were imposed onto seedlings as a combined treatment. The objective was to detect the combined effects of spectra and CN-additive on bioactive compounds and their associations with C and N concentrations in *E*. *senticosus*. According to current findings, we hypothesized that: (1) bioactive compound contents were higher in the spectrum with high red-light and low green-light, (2) CN addition can increase bioactive compounds in *E*. *senticosus*, and (3) bioactive compounds had negative associations with C and N. Our results will contribute to *E*. *senticosus* development and provide new evidence for medicinal plant quality evaluation in plant factories.

## Materials and methods

### Plant material and growth condition

*E*. *senticosus* seeds were obtained from an orchard in the central part of Heilongjiang province (45°37’ N, 31°15’ E) and transported to Heilongjiang University of Chinese Medicine (45°43’ N, 126°38’) on ice (0–3.8°C). Seeds were then prepared in sands, sterilized by potassium permanganate (0.5%, w/w), and sown and germinated in sands (moisture over 60%) in spring of 2018. Germinated seedlings were moved to plastic pots (top diameter × height, 12cm × 12cm), which was filled with mixed peat and perlite (3:1, v/v, pH adjusted to 6.0). Potted seedlings were placed in tanks (57cm length, 37cm width, 9cm height) with a water table at 3–4cm. This waters seedlings by a sub-irrigation system through rhizosphere uptake [18, 45]. Sub-irrigation waters plants by delivering water to leaves through capillary pore uptake through the plant root zone. A total of 12 pots were placed in a tank with even spacing and raised in the greenhouse on campus for one month. Then, seedlings were moved to three cultural benches. Ventilation was used to promote air flow to maintain temperatures at 18/26°C (night/day) and relative humidity (RH) at 70–80% throughout the experiment. Seedlings were shaded from sunlight for necessary conditions, which created a background illumination of 7000–23000 lux.

### Artificial lighting treatment

**Fig 1:**
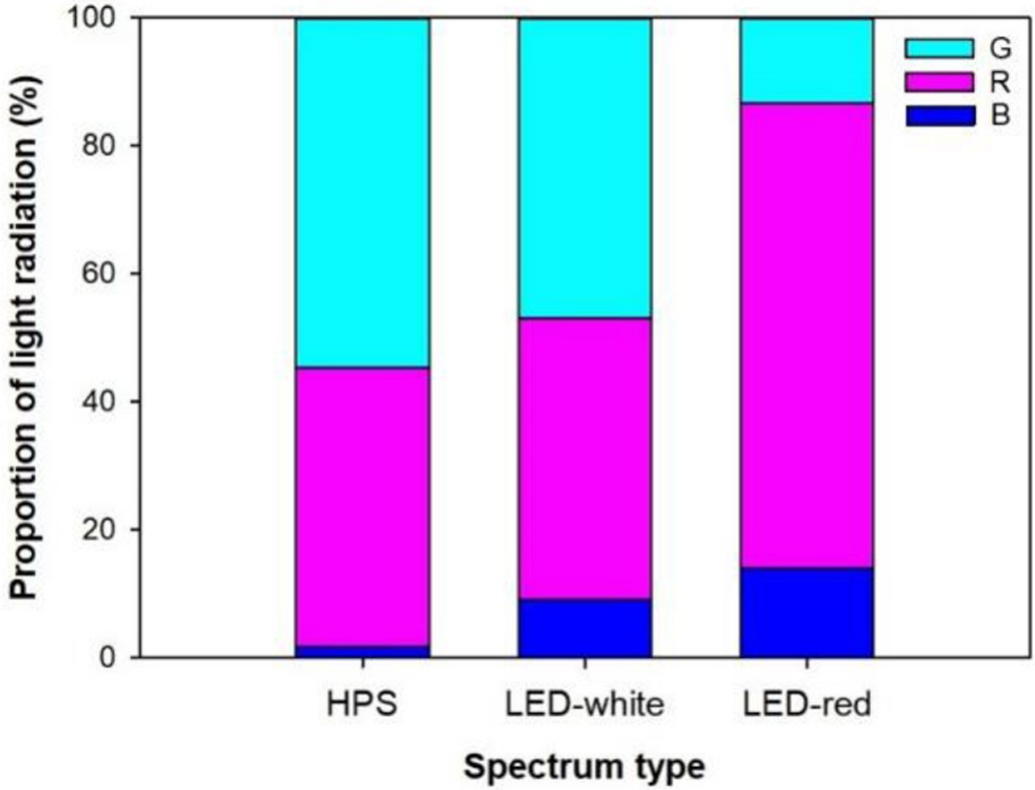
Proportional of light radiations in photosynthetic photon flux density for spectra emitted from high-pressure sodium (HPS) lamps and light-emitting diode (LED) panels. LED spectra further include white-light and red-light colors generated by combined proportions of green (G), red (R), and blue (B) light wavelengths.

Seedlings were exposed to three types of artificial lighting treatments. HPS lamps emitted a spectrum with about a 40% red light wavelength, which was similar to the white-light LED spectrum (Fig 1). The red-light LED spectrum emitted a spectrum with a higher proportion of red light but a lower proportion of green light. LED panels were placed 80cm above the bench surface. Two tanks of potted seedlings were placed together under a LED panel. Photosynthetic photon flux density (PPFD) was measured to be 93.6–94.8 μmol m^-2^ s^-1^ (depending on the type of LED spectra) ~35 cm beneath the diodes. Two HPS lamps were hanged over the other pair of tanks with one lamp per tank. HPS lamps were placed at a height such that potted seedlings were subjected to a similar PPFD to those exposed to LED spectra. Lights were turned on from 05:00 am to 21:00 pm.

### Chitosan nanoparticle addition synthesis and application

Ionotropic gelation was used to synthesize CN primary products [46, 47]. Briefly, CO was dissolved in distilled water into an aqueous solution (2.6 mg mL^-1^) with acetic acid (0.175 M) (AS1). Sodium tripolyphosphate solution (0.6 mg mL^-1^) was added dropwise to the suspension of AS1 in a volumetric proportion of 1:3. The mixture was stirred for 45 min at 25°C, cooled to room temperature as a CN reagent, and sealed in ice until being delivered to the seedlings.

Half of the potted seedlings were treated with the CN addition and the other half only received distilled water at the same volume. A volume of 10 mL CN was delivered to the surface of the growing media through a 5-mL pipettor. Delivery avoided contact with plants to prevent contamination from entering through stem cells [48, 49]. All seedlings were watered through a sub-irrigation system where leaching was mostly avoided from substrates [41]. All seedlings received nutrient supply with a formulation of Song et al. [50], which consisted of 10% N, 4% P_2_O_5_, and 7% K_2_O at a total rate of 60 mg N plant^-1^. A tank of eight seedlings was the basic unit for a combined treatment (spectra × CN) and every treatment was replicated three times. Seedlings were cultured for four months and sampled.

### Sampling, measure, and determination

All seedlings were sampled and measured for their height (length of stem) and RCD (stem diameter 2cm above the scar-dividing-line). Four seedlings per tank were sampled for dry mass and subsequent parameters and the other four were sampled for parameters determined using fresh samples. All sampled seedlings were divided into shoot and root parts and shoots were further excised into leaves and stems. The fresh weight of leaves, stem, and root were measured, then samples were dried in an oven at 70°C for 72h. Dried samples were measured for their weight again; thereafter the *WCR* can be calculated as [50, 51]:

$$WCR=\frac{W_{1}-W_{2}}{W_{1}}$$ (1)

where *W*_1_ is the mass weight before oven-drying (fresh) and *W*_2_ is the mass after drying. Dried samples were milled to powers, and 10mg of power were used to determine C and N concentrations with an element analyzer (EA-3000, Boaying Tech., Shanghai, China) [52]. Fresh samples were used to determine the contents of bioactive compounds. High performance liquid chromatography (HPLC) equipped to a Welchrom C18 chromatographic column (250mm × 4.6mm, 5 μm) was used as the instrument. Flowing phase A consisted of 0.1% acetonitrile as an aqueous solution and mobile B consisted of 0.1% phosphoric acid solution. Flowing samples were eluted using a gradient methodology, whose spectrum consisted of a gradient of concentrations in HPLC (Table 1).

**Table 1:** Gradient concentrations of phases A and B in HPLC.

Time (min)	Phase A (acetonitrile)	Phase B (phosphoric acid solution)
0	8	92
8	8	92
15	10	90
25	10	90
30	15	85
45	15	85
55	40	60

**Fig 2:**
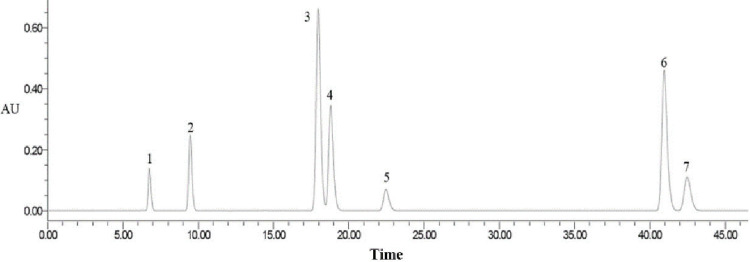
HPLC chromatograms of mixed standards. 1. L-PHE; 2. protocatechuic acid; 3. eleutheroside B; 4. chlorogenic acid; 5. caffeic acid; 6. eleutheroside E; 7. isofraxidin.

The reference was created using a mixture of standard reagents of 0.462 mg L-PHE, 4.615 mg protocatechuic acid, 100.692 mg chlorogenic acid, 3.952 mg eleutheroside B, 3.261 mg eleutheroside E, 3.461 mg caffeic acid, and 0.769 mg isofraxidin. The ion chromatograms for standard references of mixed standard reagents are shown in Fig 2. For determining the sample, a 2.0g sample was moved to a 100 mL conical flask where 50 mL methyl alcohol (70%, v/v) was added. The mixture was extracted with ultrasound (250W and 50kHz) four times every 30 min. Supernatants were filtered and diluted to 3 mL with methyl alcohol (70%, v/v).

### Statistical analysis

Data were analyzed by a split-block design, where the main block was spectra (HPS, red LED, white LED) and the sub-block was CN addition (with vs without). The random factor was assigned as the placement of blocks, which was replicated three times. Analysis of variance (ANOVA) was used to detect their interactive effects on plant growth, mass accumulation (fresh and dry), WCR, C and N concentrations, and bioactive compound contents. When significant effects were detected, means were compared by Turkey test to identify significant difference at 0.05 level. Caffeic acid showed no response to any treatments in this study; hence it was excluded from further analysis. Principal component (PC) analysis was used to detect for interrelationships among parameters. Trends of correlations were characterized by eigenvalues along axes of PC-1 and PC-2.

## Results

### Seedling growth

**Fig 3:**
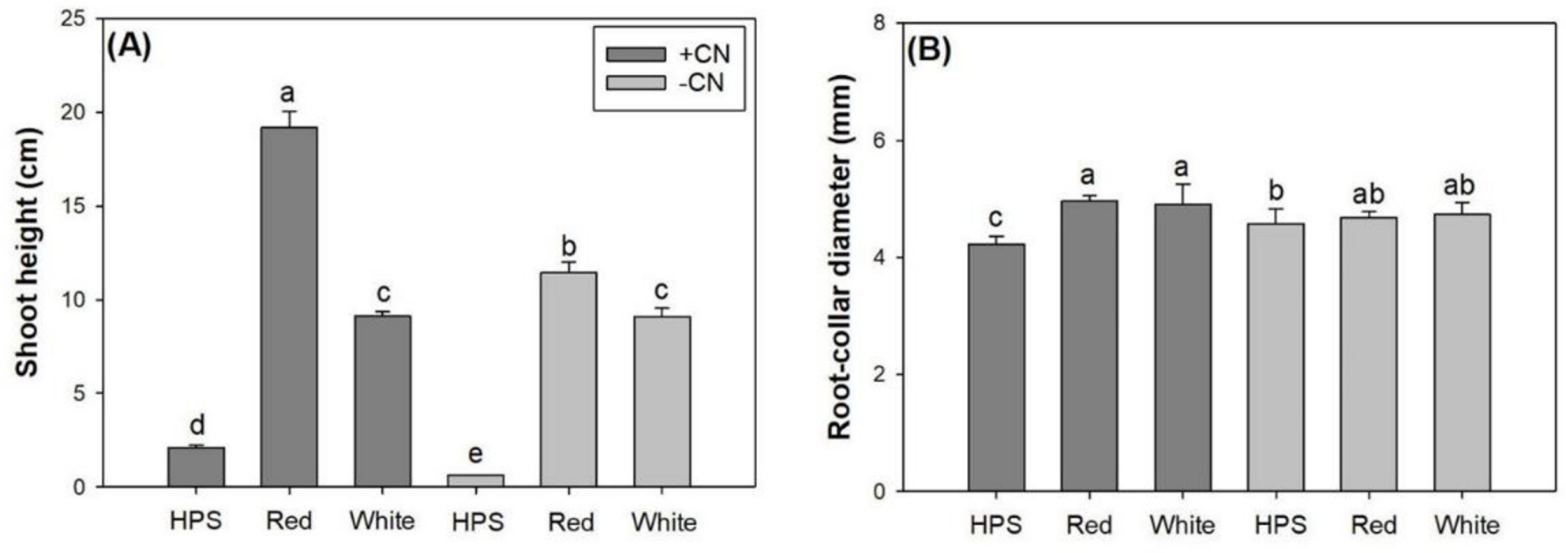
Shoot height (A) and root-collar diameter (B) in *Eleutherococcus senticosus* seedlings subjected to combined spectra and chitosan nanoparticle addition (CN) treatments. + CN, with CN addition; -CN, without CN addition; HPS, high-pressure sodium spectrum; Red, red-light light-emitting diode (LED) spectrum; White, white-light LED spectrum. Error bars present standard errors and different letters are given over means with differences identified by Turkey test (α<0.05).

Light spectra and CN addition had an interactive effect on shoot height and root-collar diameter (RCD) in *E*. *senticosus* seedlings (Table 2). Shoot height was taller under the light-emit diode (LED) spectra compared to that under the high-pressure sodium (HPS) regardless of whether CN was added or not (Fig 3A). The red-light spectrum resulted in higher shoot height than the white-light spectrum with or without CN additions. The CN addition increased shoot height in seedlings subjected to the HPS or to the red-light spectra. For seedlings exposed to the white-light spectrum, CN addition did not result in a difference in shoot height. The LED spectra only increased RCD for seedlings receiving CN addition (Fig 3B). For seedlings that received the CN addition, there were no difference in RCD between the two LED spectra treatments.

**Table 2:** *F* values from analysis of variance (ANOVA) of effects of light quality (Light), chitosan nanoparticle addition (CN), and their interaction (Light × CN) on aerial growth and whole-plant dry weight, fresh weight, and water content ratio in *Eleutherococcus senticosus* seedlings.

Seedling parameters	Light	CN	Light × CN
Shoot height	3461.54*** ^1^	505.75***	298.91***
RCD ^2^	21.75***	0.37	10.00**
Dry weight in leaves	844.05***	438.69***	50.82***
Dry weight in stem	919.92***	1777.19***	294.81***
Dry weight in root	1439.32***	679.81***	22.99***
R/S ^3^	370.22***	3.14	20.62***
Fresh weight in leaves	1286.14***	591.71***	130.82***
Fresh weight in stem	261.72***	653.96***	139.24***
Fresh weight in root	862.38***	175.78***	0.57
WCR ^4^ in leaves	66.99***	24.36***	9.91***
WCR in stem	42.58***	1.33	5.17**
WCR in root	8.18***	65.57***	9.19***

^1^ Number of asterisks indicates levels of significances depending on *P* values:

**, *P*<0.01;

***, *P*<0.001;

^2^ RCD, root-collar diameter;

^4^ WCR, water content ratio.

### Mass accumulation and alloction and water content

**Fig 4:**
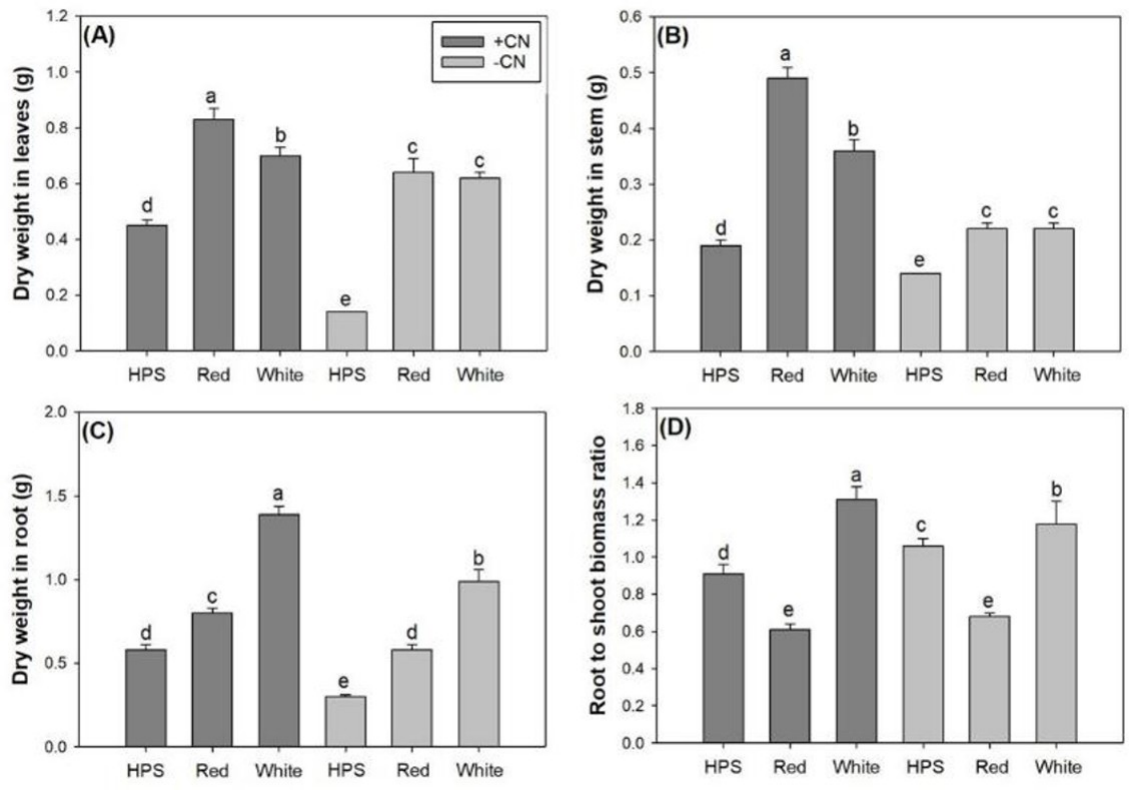
Dry weight in leaves (A), stem (B) and root (C) and root to shoot biomass ratio (D) in *Eleutherococcus senticosus* seedlings subjected to combined spectra and chitosan nanoparticle addition (CN) treatments. + CN, with CN addition; -CN, without CN addition; HPS, high-pressure sodium spectrum; Red, red-light light-emitting diode (LED) spectrum; White, white-light LED spectrum. Error bars present standard errors and different letters are given over means with differences identified by Turkey test (α<0.05).

Light spectra and CN addition also had an interactive effect on biomass accumulation and allocation in *E*. *senticosus* seedlings (Table 2). The LED spectra increased dry-mass production in leaves, stem, and root compared to the HPS spectrum (Fig 4A–4C). For shoot parts, the red-light LED spectrum with CN addition treatment resulted in greater dry mass weight in the leaves and stem, and there was no difference in dry mass weight in seedlings without CN addition between the two LED spectra (Fig 4A and 4B). The CN addition increased dry mass weight in the leaves and stem of seedlings subjected to both HPS and LED spectra. For the root part, the white-light LED spectrum resulted in greater dry mass weight compared to the red-light spectrum with or without CN additions (Fig 4C). The addition of CN increased dry mass weight in the root for all spectra treatments. Root to shoot mass ratio (R/S) was lower in seedlings subjected to the red-light LED spectrum compared to those subjected to the HPS, and both were lower compared to those subjected to the white-light spectrum (Fig 4D). The CN addition decreased R/S in seedlings subjected to the HPS spectrum but increased R/S in seedlings subjected to the white-light LED spectrum (Fig 4D).

**Fig 5:**
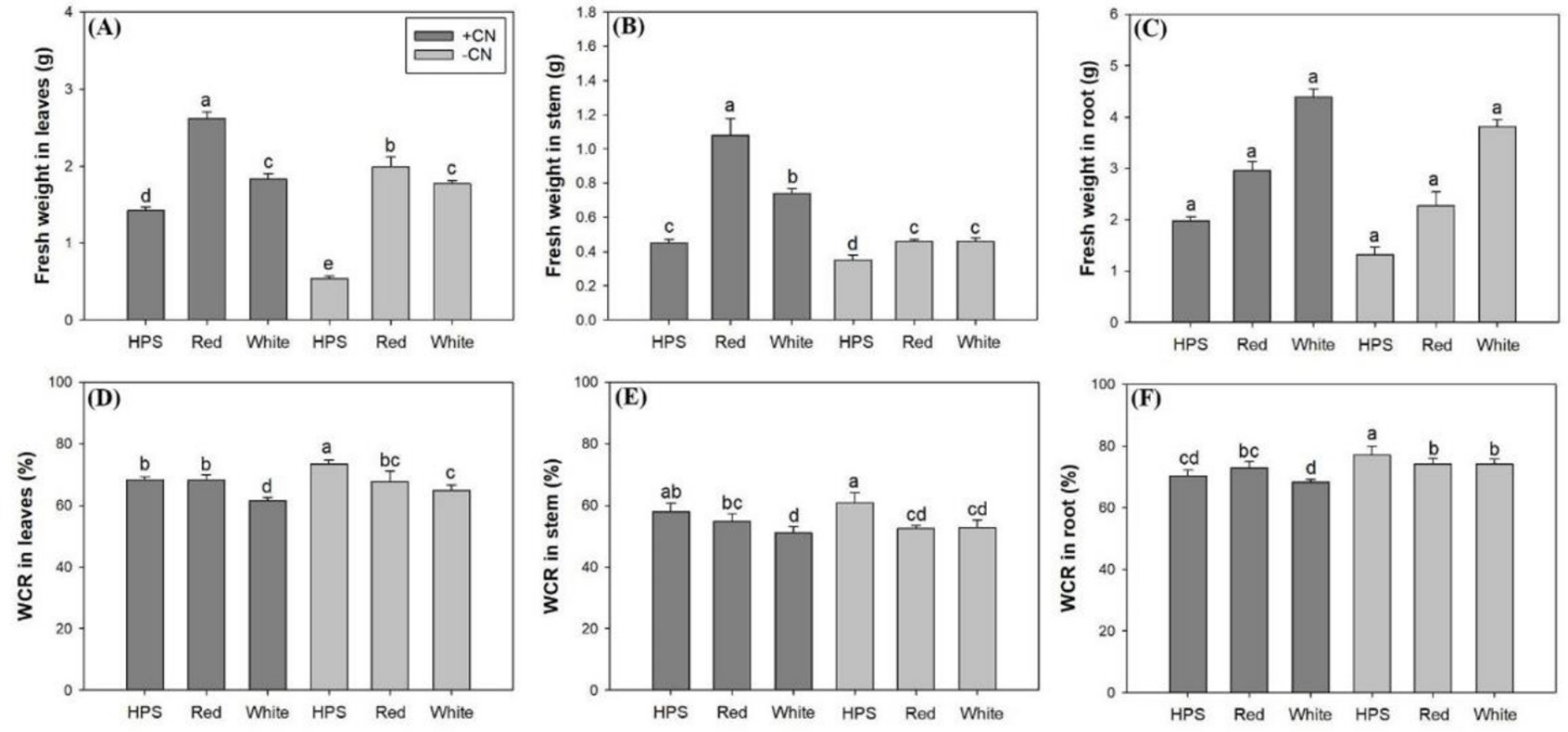
Fresh weight in leaves (A), stem (B) and root (C) and water content ratio (WCR) in these three parts (D, E, F, respectively) in *Eleutherococcus senticosus* seedlings subjected to combined spectra and chitosan nanoparticle addition (CN) treatments. + CN, with CN addition; -CN, without CN addition; HPS, high-pressure sodium spectrum; Red, red-light light-emitting diode (LED) spectrum; White, white-light LED spectrum. Error bars present standard errors and different letters are given over means with differences identified by Turkey test (α<0.05).

Fresh mass was impacted by the interaction between light spectra and CN addition for the leaf and stem, but there was not a significant impact for the root (Table 2). The LED-spectra resulted in greater fresh mass weight in the leaves and stem compared to the HPS spectrum (Fig 5A and 5B). The red-light spectrum resulted in greater fresh mass weight in the leaves and stem compared to the white-light spectrum, but difference in the fresh mass weight of the stem was not significant between the two LED spectra (Fig 5B). In shoot parts, CN addition increased fresh mass weight in most spectra except for the leaves of seedlings exposed to the white-light LED spectrum. For roots, the lighting spectra had a significant main effect (Table 2) in inducing greater fresh mass weight in seedlings exposed to the red-light spectrum (2.62±0.37 g) and white-light spectrum (4.11±0.29 g) relative to those exposed to the HPS spectrum (1.65±0.31 g) (Fig 5C). The CN addition also resulted in greater fresh mass weight in roots (3.11±0.86 g and 2.47±0.90 g for CN and control, respectively).

Light spectra and CN addition also had an interactive effect on water content ratio (WCR) in *E*. *senticosus* seedlings (Table 2). WCR in shoot parts was decreased by exposure to the white-light LED spectrum relative to the HPS spectrum, but difference from expoure to the red-light spectrum was not significant (Fig 5D and 5E). In seedlings with the CN addition treatment, the red-light spectrum resulted in higher RCR in the leaves and stem compared to the white-light spectrum. However, in the roots, there was no difference in WCR between LED and HPS spectra for CN-treated seedlings. For the no CN addition treatment, LED-exposed seedlings had lower WCR compared to HPS-exposed seedlings (Fig 5F).

### Carbon and nitrogen concentrations

Light spectra and CN addition had no interactive effects on carbon (C) concentration in any seedling organ; but they had an interactive effect on nitrogen (N) concentration in shoot parts and either light or CN addition had a separately main effects on root N concentration (Table 3).

**Table 3:** *F* values from analysis of variance (ANOVA) of effects of light quality (Light), chitosan nanoparticle addition (CN), and their interaction (Light × CN) on nitrogen (N) and carbon (C) concentrations in *Eleutherococcus senticosus* seedlings.

Seedling parameters	Light	CN	Light × CN
Leaf C	1.11	0.25	1.74
Stem C	5.45* ^1^	29.11***	1.41
Root C	9.00**	15.04**	4.86*
Leaf N	2.79	0.80	5.55*
Stem N	130.74***	553.09***	14.03***
Root N	90.54***	124.22***	2.64

Note: ^1^ * significance: *P*<0.05.

**Fig 6:**
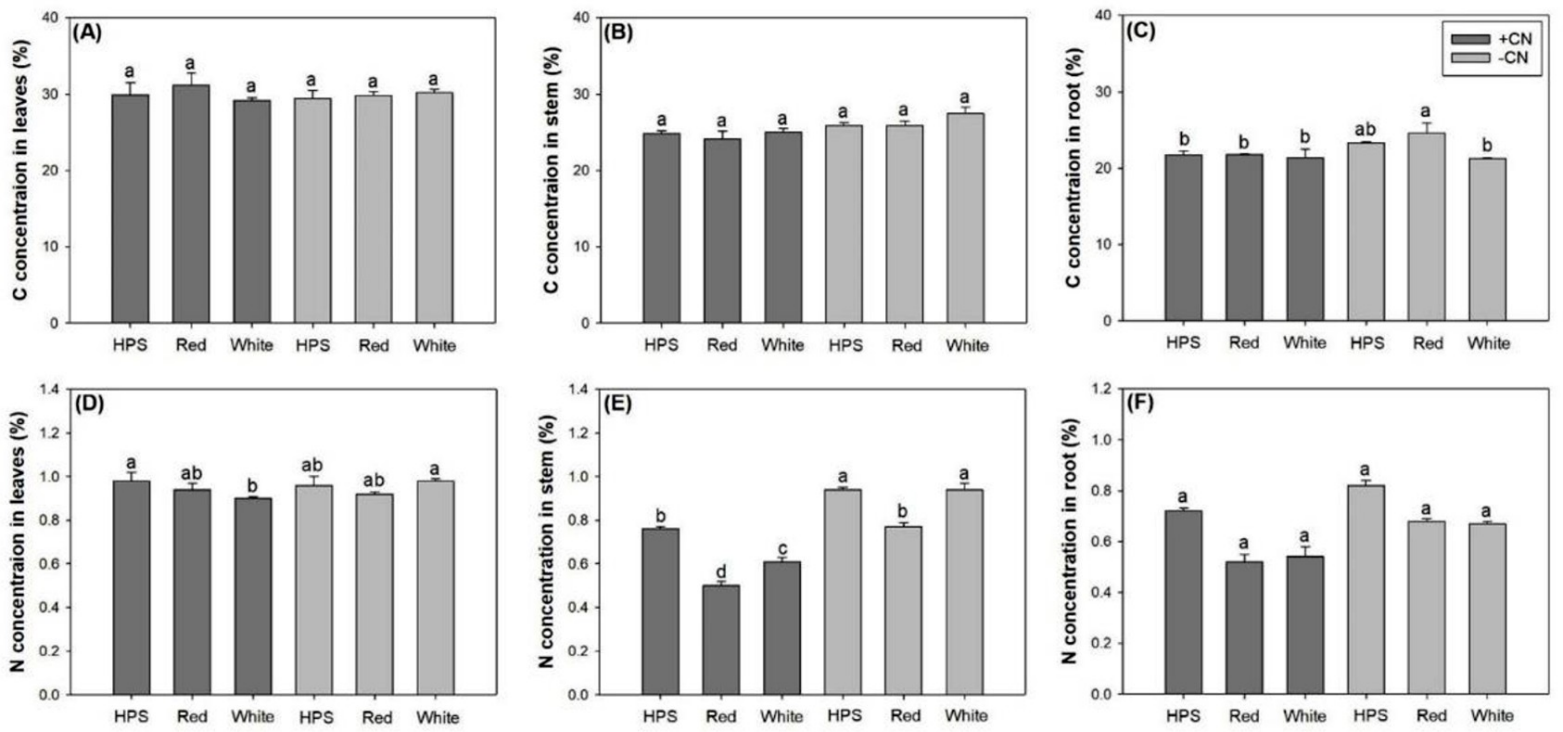
Carbon (C) concentration in leaves (A), stem (B) and root (C) and nitrogen (N) in these three parts (D, E, F, respectively) in *Eleutherococcus senticosus* seedlings subjected to combined spectra and chitosan nanoparticle addition (CN) treatments. + CN, with CN addition; -CN, without CN addition; HPS, high-pressure sodium spectrum; Red, red-light light-emitting diode (LED) spectrum; White, white-light LED spectrum. Error bars present standard errors and different letters are given over means with differences identified by Turkey test (α<0.05).

Neither light spectra or CN had any significant effects on C concentration in leaves (Fig 6A). Leaf C concentration ranged between 28–32% in leaves and 24–27% in stem (Fig 6A and 6B). Stem C concentration in seedlings subjected to the HPS spectrum (25.38±0.62%) was not statistically different from that in seedlings subjected to red- (25.00±1.11%) or white-light spectra (26.26±1.34%). However, stem C concentration under white-light spectrum was higher than that under red-light. The CN addition decreased stem C concentration by 6.55% (24.68±0.65% vs 26.41±0.88% with vs without CN additions, respectively). Seedlings with CN additions did not show any difference in C concentration in the roots among spectra (Fig 6C). However, in seedlings without CN addition, the red-light LED spectrum resulted in higher C concentration in the roots compared to the white-light spectrum, but both had no significant difference from root C concentration in the HPS treatment. The CN addition decreased root C concentration only in seedlings exposed to the red-light spectrum.

Light spectra and CN addition had an interactive effect on N concentration in the leaves and stem (Fig 6D and 6E), but they had no impact on root N concentration (Fig 6F). Compared to the HPS spectrum, the white-light LED spectrum had lower N concentration in the leaves and stem of seedlings that received the CN addition (Fig 6D and 6E). The red-light spectrum also had lower stem N concentration compared to the HPS spectrum regardless of CN addition (Fig 6E). Root N concentration was lower in the red-light (0.60±0.09%) and white-light spectra (0.61±0.08%) compared that in the HPS spectrum (0.77±0.05%) (Fig 6F). The CN addition decreased root N concentration by 18.20% (0.59±0.09% vs 0.73±0.07% with vs without CN addition, respectively).

### Bioactive compound contents

**Fig 7:**
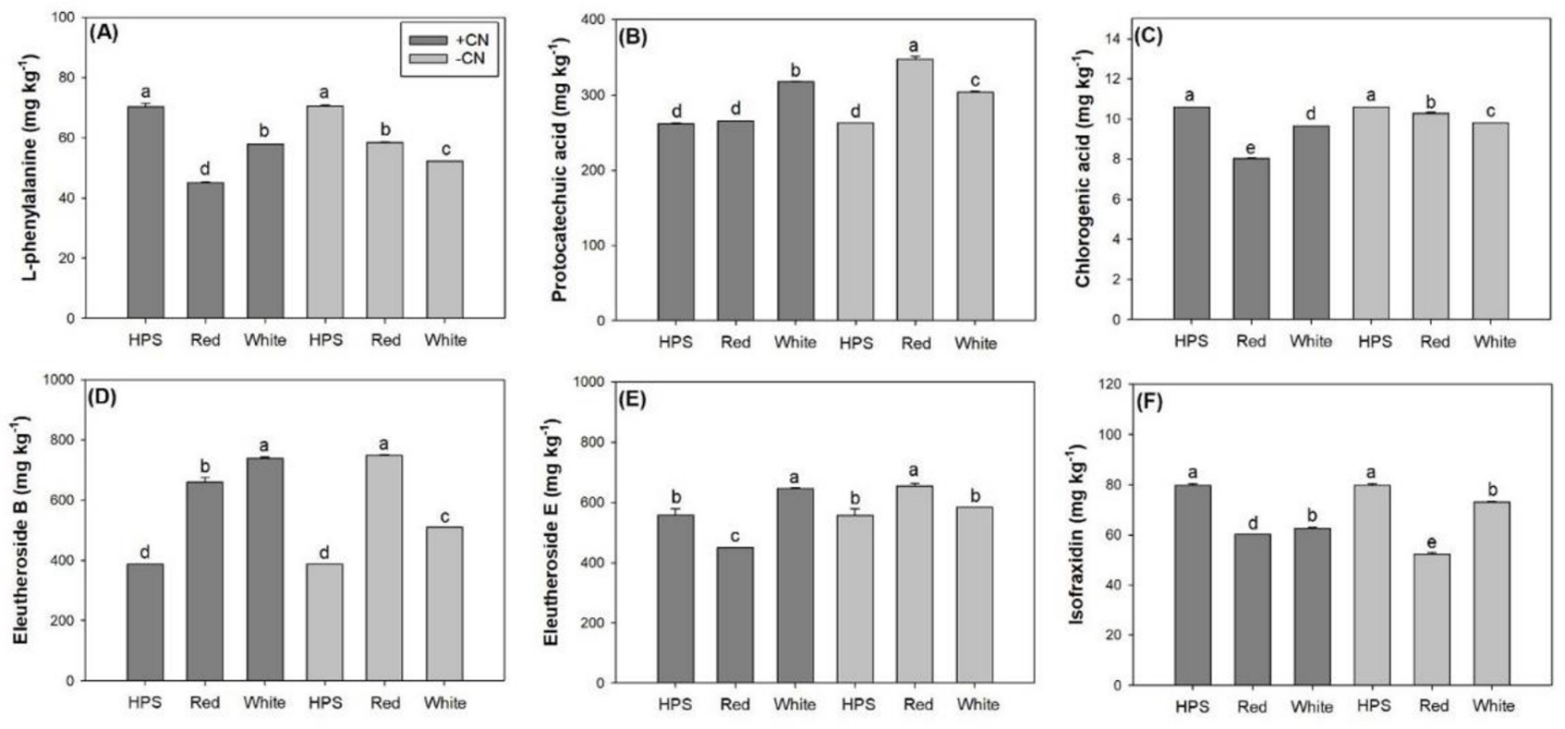
Contents of L-phenylalanine (A), protocatechuic acid (B), chlorogenic acid (C), eleutheroside-B (D), eleutheroside-E (E), and isofraxidin (F) in stems of *Eleutherococcus senticosus* seedlings subjected to combined spectra and chitosan nanoparticle addition (CN) treatments. + CN, with CN addition; -CN, without CN addition; HPS, high-pressure sodium spectrum; Red, red-light light-emitting diode (LED) spectrum; White, white-light LED spectrum. Error bars present standard errors and different letters are given over means with differences identified by Turkey test (α<0.05).

Light spectra and CN addition had an interactive effect on the contents of all types of bioactive compounds in the stems of *E*. *senticosus* seedlings (Table 4). For aerial plant parts, the LED spectra resulted in lower L-phenylalanine (L-PHE) content in the stem compared to the HPS spectrum (Fig 7A). Compared to the white-light LED spectrum, the red-light spectrum with CN addition had lower L-PHE content in the stems but had higher L-PHE content without CN addition. The white-light LED spectrum increased protocatechuic acid content in the stems of seedlings with or without CN additions, but the red-light spectrum only increased protocatechuic acid content in seedlings without CN addition (Fig 7B). The LED-light spectra decreased chlorogenic acid content in the stem compared to the HPS spectrum (Fig 7C). The red-light spectrum decreased chlorogenic acid content in the stem compared to the white-light spectrum in seedlings with CN addition, but results were inverse in seedlings without CN addition. The LED-light spectra increased eleutheroside B content in the stem compared to the HPS spectrum, and, again, the red- and white-light LED spectra resulted in contrasting responses for seedlings subjected to CN addition or to water (Fig 7D). The red-light LED spectrum decreased eleutheroside E content in the stems of seedlings with CN addition compared to the HPS spectrum, while the white-light spectrum increased eleutheroside E content (Fig 7E). For seedlings without CN addition, compared to the HPS spectrum, the white-light spectrum did not impact eleutheroside E content but the red-light spectrum increased eleutheroside E content. Stem isofraxidin content was lower in seedlings exposed to the LED spectra instead of the HPS spectrum, but it was higher in seedlings exposed to the white-light spectrum instead of the red-light spectrum (Fig 7F).

**Table 4:** *F* values from analysis of variance (ANOVA) of effects of light quality (Light), chitosan nanoparticle addition (CN), and their interaction (Light × CN) on bioactive compounds in stems and roots of *Eleutherococcus senticosus* seedlings.

Seedling parameters	Light	CN	Light × CN
Stem: L-PHE ^1^	1790.31***	92.05***	435.68***
Stem: protocatechuic acid	1472.92***	810.65***	1347.23***
Stem: chlorogenic acid	4896.59***	4506.28***	3700.89***
Stem: eleutheroside-B	3636.39***	216.30***	901.42***
Stem: eleutheroside-E	40.27***	56.28***	162.61***
Stem: isofraxidin	3195.80***	14.29**	489.58***
Root: L-PHE	7810.34***	364.34***	83.31***
Root: protocatechuic acid	12254.60***	1182.92***	906.85***
Root: chlorogenic acid	41796.3***	13935.80***	6018.16***
Root: eleutheroside-B	910.30***	13296.40***	31352.4***
Root: eleutheroside-E	3278.99***	1326.55***	2631.86***
Root: isofraxidin	2728.28***	233.42***	78.03***

^1^ PHE, phenylalanine.

**Fig 8:**
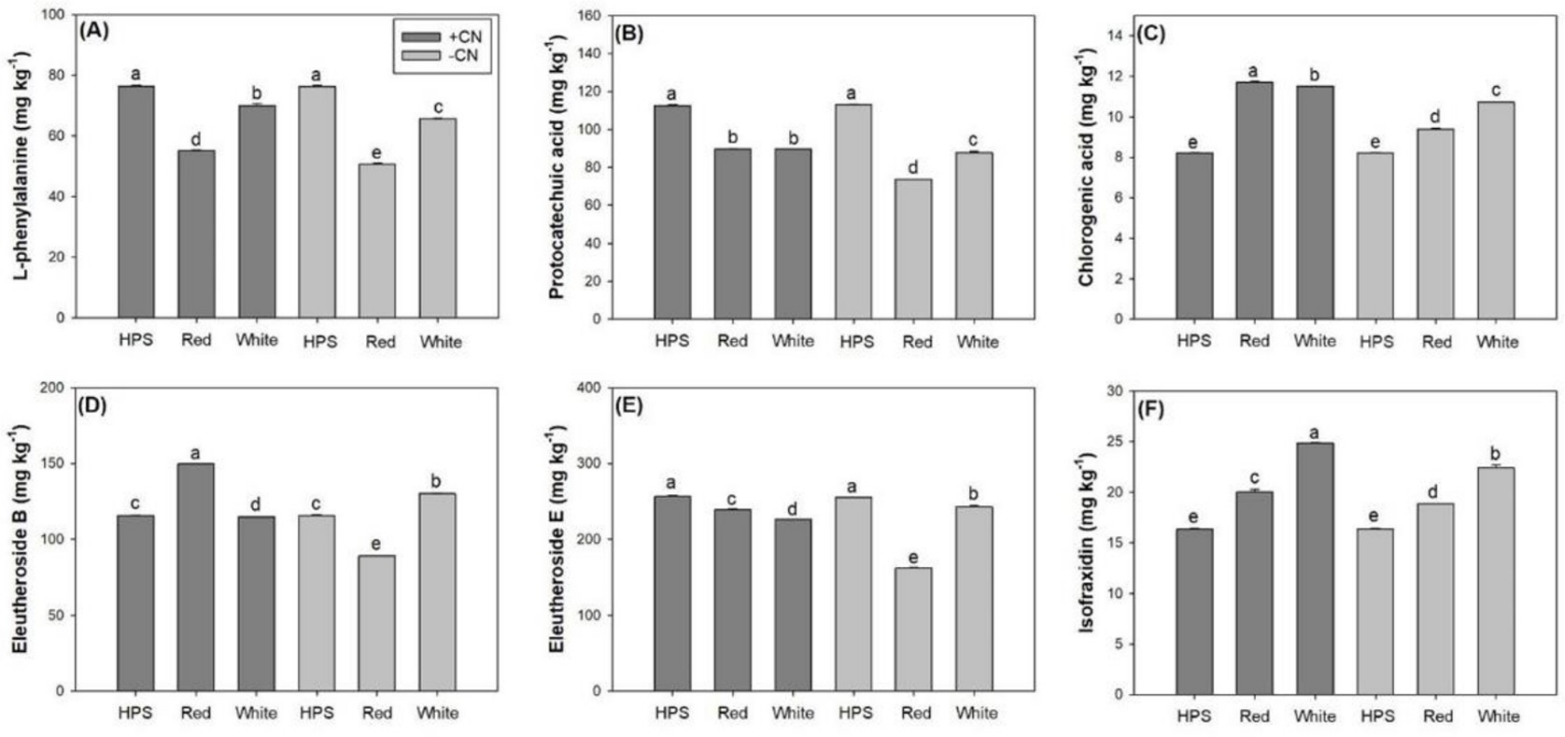
Contents of L-phenylalanine (A), protocatechuic acid (B), chlorogenic acid (C), eleutheroside-B (D), eleutheroside-E (E), and isofraxidin (F) in roots of *Eleutherococcus senticosus* seedlings subjected to combined spectra and chitosan nanoparticle addition (CN) treatments. + CN, with CN addition; -CN, without CN addition; HPS, high-pressure sodium spectrum; Red, red-light light-emitting diode (LED) spectrum; White, white-light LED spectrum. Error bars present standard errors and different letters are given over means with differences identified by Turkey test (α<0.05).

Light spectra and CN addition also had an interactive effect on the contents of all types of bioactive compounds in the roots of *E*. *senticosus* seedlings (Table 4). The LED-light spectra decreased root L-PHE content compared to the HPS spectrum and between the LED-light spectra, the red-light spectrum decreased root L-PHE content more (Fig 8A). The LED-light spectra also decreased protocatechuic acid content in the roots of seedlings compared to the HPS spectrum (Fig 8B). Compared to the white-light spectrum, the red-light spectrum decreased stem protocatechuic acid content in seedlings without CN addition but did not impact seedlings with CN addition. Chlorogenic acid content in the roots was increased by the LED-light spectra relative to the HPS spectrum (Fig 8C). In seedlings with CN addition, the red-light spectrum resulted in higher root chlorogenic acid content compared to the white-light spectrum, and in seedlings without CN addition, the red-spectrum decreased root chlorogenic acid content. The two LED-light spectra resulted in contrasting eleutheroside B contents relative to the HPS spectrum (Fig 8D). The red-light spectrum resulted in higher root eleutheroside B content in seedlings with CN addition and lower root eleutheroside B content in seedlings without CN addition compared to the white-light spectrum. The LED-light spectra decreased eleutheroside E content in the roots compared to the HPS spectrum (Fig 8E). Relative to the white-light spectrum, root eleutheroside E content was higher in seedlings with CN addition but was lower in seedlings without CN addition when exposed to the red-light spectrum. Root isofraxidin content was increased by the LED-light spectra compared to the HPS spectrum (Fig 8F). Red-light spectrum further decreased root isofraxidin content relative to the white-light spectrum.

### Association of bioactive compounds and C and N concentrations

**Fig 9:**
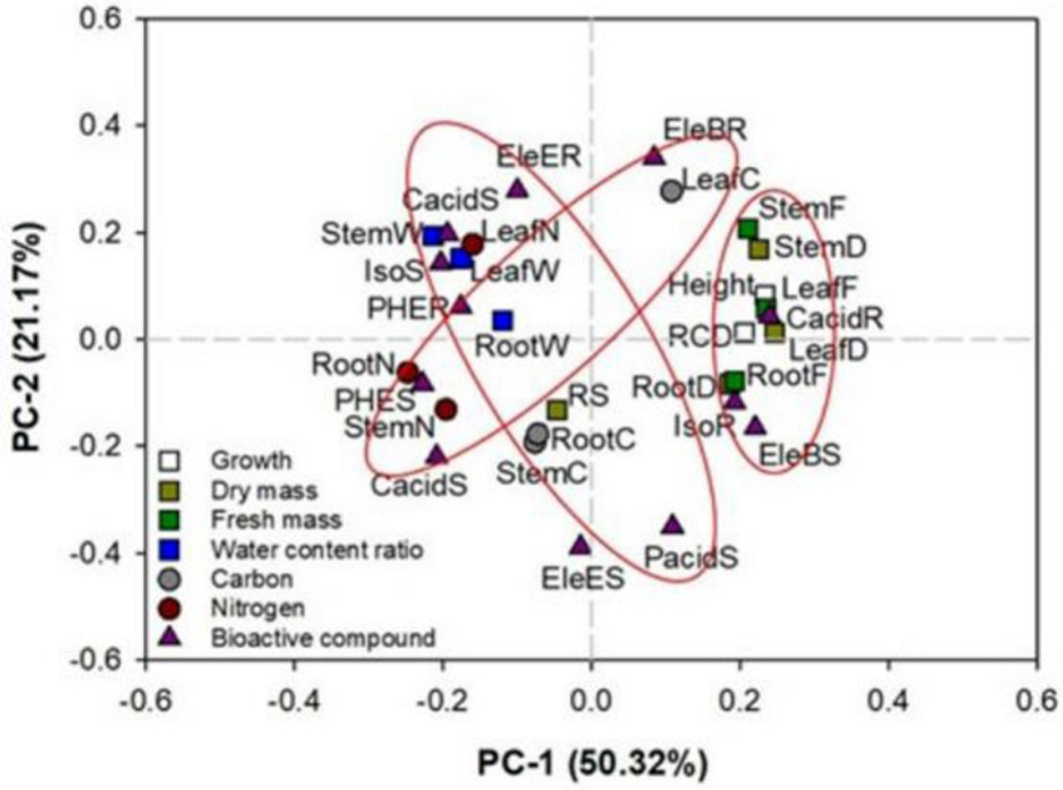
Principal component (PC) analysis of parameters about growth, dry mass, fresh mass, WCR, C concentration, N concentration, and bioactive compound contents in shoots and roots of *Eleutherococcus senticosus* seedlings subjected to combined spectra and chitosan nanoparticle addition treatments. Height, shoot height; RCD, root-collar diameter; LeafD, leaf dry mass weight; StemD, stem dry mass weight; RootD, root dry mass weight; RS, root to shoot dry mass ratio; LeafF, leaf fresh mass weight; StemF, stem fresh mass weight; RootF, root fresh mass weight; LeafW, leaf WCR; StemW, stem WCR; RootW, root WCR; LeafC, leaf C concentration; StemC, stem C concentration; RootC, root C concentration; LeafN, leaf N concentration; StemN, stem N concentration; RootN, root N concentration; PHES, stem phenylalanine content; PacidS, stem protocatechuic acid content; CacidS, stem chlorogenic acid; EleBS, stem eleutheroside-B content; EleES, stem eleutheroside-E content; IsoS, stem isofraxidin content; PHER, root phenylalanine content; PacidR, root protocatechuic acid content; CacidR, root chlorogenic acid; EleBR, root eleutheroside-B content; EleER, root eleutheroside-E content; IsoR, root isofraxidin content.

The principal component (PC) analysis reveals a total of 71.49% in data variation, where the first PC (PC-1) accounted for 50.32% and the second (PC-2) accounted for 21.17% (Fig 9). Factors such as growth, mass weight (fresh and dry), and parts of bioactive compounds had eigenvalues to a similar level along the axis of PC-1. Specifically, seedling height, RCD, fresh and dry mass weight in the stem and root, eleutheroside-B in the stem, and chlorogenic acid and isofraxidin in the root had a positive association, but a negative association with root N concentration. Eleutheroside-B in the root had a positive association with leaf C concentration, and both were negatively associated with N concentration in the stem and root and L-PHE and chlorogenic acid in the stem. Protocatechuic acid in the stem was also negatively associated with eleutheroside-E in the root, chlorogenic acid in the stem, and leaf N concentration.

## Discussion

### Plant growth, mass production, and water content

With the HPS spectrum as reference, the red-light LED spectrum promoted growth (both in stem length and diameter thickening) and dry- and fresh-mass allocation (either in leaves or in stem) in aerial organs of *E*. *senticosus* seedlings. Although the red-light spectrum increased dry mass in the root compared to the HPS spectrum, the proportion of dry mass in root was lower than the proportion in the shoot and R/S was lowered by the red-light spectrum. In addition, the red-light spectrum does not promote water uptake in the root nor promote fresh weight in the root. However, the red-light LED spectrum (over 40% red light in wavelength) did not significantly affect R/S in tree seedlings, such as in trials on *Ormosia hosiei* [53] and *Pinus koraiensis* [19]. In contrast, the white-light spectrum not only promoted whole-plant (shoot plus root) growth and dry mass accumulation, but it also increased R/S compared to the HPS spectrum. Our white-light LED spectrum had a similar proportion of red, green, and blue lights as the HPS spectrum. The promotion of R/S given exposure to the white-light LED spectrum was found in our study. Similar findings were also reported on *Podocarpus macrophyllus* [20]. The white-light LED spectrum caused lower fresh and dry weight and water content in the shoots compared to the red-light LED spectrum; the white-light LED spectrum did not promote as efficient water uptake which limited fresh weight. Overall, both types of LED spectra promoted increased mass relative to the HPS spectrum but in different ways. The red-light spectrum was effective in promoting water uptake but not in dry mass allocation to the roots, while the white-light spectrum was the opposite. The response of seedlings to these effects were not impacted by the CN treatment.

### Carbon and nitrogen cycling

The HPS spectrum did not significantly change C concentration compared to the LED spectra and different C changes mainly occurred between the two types of LED spectra. A lower C concentration in the stems of *E*. *senticosus* seedlings exposed to the red-light spectrum was due to the cost of water uptake [54]. The addition of chitosan oligosaccharide (CO) was reported to enhance starch allocation to promote root resistance [29]. This and the red-light spectrum interacted to increase root C concentration. The white-light spectrum probably lowered root C concentration through the consumption of carbohydrate for synthesizing bioactive compounds. Lower root C concentration in LED light with less red-light wavelength was also reported in *Allium victorialis* [26], which, along with our findings, suggests that one effect of seedlings being exposed to white LED light is increased C usage.

In *E*. *senticosus* seedlings, the LED-light spectrum led to the decline of N concentration in shoot organs, especially when interacting with CN addition. This was because increased mass production and allocation consumes more N compounds but there was no additional N inputs [51, 55, 56]. In another Araliaceae-family plant, *Aralia elata*, LED spectrum also resulted in the decline of N concentration and the decline in shoots was higher than that in roots [4]. This was identified as N dilution induced by stimulated growth [20, 42, 44]. Stem N concentration was lower in the red-light spectrum than in the white-light spectrum, which was the result of greater mass allocation and its consequence of N dilution. The CN addition had a similar effect which reduced N concentration by stimulating mass production and promoting dilution. This effect of CN addition concurred with the effect of CO addition to *P*. *macrophyllus* [30] and *Eustoma grandiflorum* [57]. The interactive effects of CN addition and LED spectra resulted in even lower N concentration in the stem.

### Bioactive compound content

Our results on bioactive compound contents did not clearly support the first hypothesis. The red-light LED spectrum mostly lowered bioactive compound contents compared to the HPS spectrum except for eleutheroside B content in the stem and root and chlorogenic acid content and isofraxidin content in the root.

The white-light spectrum induced a higher level of eleutheroside B content in the stem, which agrees with findings in *E*. *senticosus* somatic embryos by Shohael et al. [58]. Although our eleutheroside E content was lower in the red-light spectrum, the inverse was reported in *E*. *senticosus* somatic embryos [58]. In our PC analysis, eigenvalues between eleutheroside-B and -E contents were plotted at generally contrasting positions which suggests discrete distributions of secondary metabolites for these two compounds. Contrasting responses of eleutheroside B and eleutheroside E to light spectra were reasonable. Eleutheroside is a lignan compound which is further divided into syringin (eleutheroside B) and (-) syringaresinol-di-O-β-D-glucoside (eleutheroside E), both of which were frequently found in *Acanthopanax* species [59].

Several pieces of evidence demonstrate that low-red-light spectrum can promote chlorogenic acid synthesis in lettuce [60], jasmonide [61], and strawberry [62]. In our study, chlorogenic acid content in stems was lower in red-light which concurs with previous findings. However, the response of chlorogenic acid content in roots to red-light was contrary. ‘Chlorogenic acid is a caffeoylquinic acid derivative known to have antioxidant activity [63].’ The upregulation of chlorogenic acid in roots is a defense mechanism to cope with underground stress when dry mass allocation is insufficient [63, 64]. Therefore, the upregulation of chlorogenic acid synthesis in roots may be a response to lower mass allocation to roots in red-light.

In our study, the red-light spectrum increased root isofraxidin content relative to the HPS spectrum, but the white light spectrum increased even more. The white light spectrum was also found to promote isofraxidin content in the roots of *Sarcandra glabra* seedlings [65]. Isofraxidin belongs to coumarin, which was also shown to be upregulated in low-red lights [66, 67]. Isofraxidin in plant root is synthesized as a bioactive response to abiotic stress. It was found that in natural *E*. *senticosus* populations, isofraxidin was strongly synthesized in roots when rainfall was limited and droughts impacted the rhizosphere [9]. Root isofraxidin synthesis was closely associated with growth and shoot and root masses, which were highly responsive to combined light and CN factors. Again, in the red-light spectrum, less dry mass was allocated to roots which likely induced the upregulation of isofraxidin.

The CN addition interacted with LED spectra and had contrasting effects on the contents of L-PHE, chlorogenic acid, eleutheroside B, and eleutheroside E. Most of these bioactive compounds were also secondary metabolites that were upregulated by the lack of dry mass allocation to roots. In the stem, the addition of CN impacted all bioactive compounds in a combination of red light exposure by switching higher levels of bioactive compound content to lower levels. According to results from *Ficus hirta* and *Alpinia xyphylla* [25], stem N concentration had a negative association with secondary metabolites. Leaf N concentration in seedlings under red-light was at a lower level without CN addition but at a higher level with CN addition. CO addition has been shown to promote N uptake, which was corroborated in our study by the interaction of CN addition with red light. Hence, the increase in N concentration may account for the decrease in bioactive compound contents in the stem. In addition, CN addition can promote resistance to stress [29]. The downregulation of bioactive compounds was also the result of enhanced resistance.

### Practical meaning of results

Our results can be used to guide culturing of *E*. *senticosus* seedlings in the plant factory environment. As a theoretical reference, our study demonstrated an instance of the adjustment of biomass production and allocation. The effect of CN addition on eleutheroside B content and C-N cyclings can be used to maximize the production of this bioactive compound for medical uses. Our study reveals that the cultivation of *E*. *senticosus* seedlings can be independent from field nursery and all aimed compounds can be produced by adjusting light spectra and CN addition.

### Limits of current study

Our study manipulated artificial lighting with different spectra. We designed the illumination environment using empirical data derived from former studies, but more precise lighting simulation should be adapted from the natural environment [6, 8]. Therefore, our first limit was managing the environment habitat of *E*. *senticosus* populations. Second, this study is the first one to detect the response of secondary metabolites in *E*. *senticosus* seedlings to CN addition; hence we did not have sufficient background information for designing the doses of CN input. Our results may be limited by the rate of CN additions to seedlings, and a wider range of doses are suggested in the future. Finally, our results were limited to an artificial plant factory condition, which can be referred to by future studies with similar settings. However, a wider spectra may be available to induce higher levels of secondary metabolites.

## Conclusions

In this study, *E*. *senticosus* seedlings were raised under exposure to three types of artificial lightings: HPS, red-light LED, and white-light LED. Half of the seedlings were treated with CN while the other half received distilled water as the control. Lighting spectra and CN addition had an interactive effect on nearly all parameters measuring the seedlings’ growth, mass production, C/N concentrations, and bioactive content. With the HPS spectrum as a reference, the red-light spectrum induced greater stem elongation and shoot dry mass accumulation with accelerated water uptake, but elicited low biomass allocated to roots. The white-light spectrum benefitted more in mass allocation in roots, maintained C concentration in stems, and alleviated N dilution. In stems, the white-light spectrum induced high protocatechuic acid with lower N concentration; in roots, the red-light spectrum resulted in higher eleutheroside B content in accordance with high N concentration. Therefore, CN addition in combination with white or red LED lights is recommended for increasing accumulation of bioactive components in the shoots and roots of *E*. *senticosus* seedlings, respectively.

## Supporting information

S1 Raw data (PDF)
