# Supplementary material for: Bioactive extracts and association with C and N in Eleutherococcus senticosus subjected to chitosan nanoparticles in contrasting light spectra
Source: PLoS One. 2022 Dec 1;17(12):e0277233. doi: 10.1371/journal.pone.0277233 (PMC9714952; doi:10.1371/journal.pone.0277233)
Supplement: S1 Raw data — (PDF) [file pone.0277233.s001.pdf]

CN	Light	Replicate	Height	RCD	DWRoot	DWStem	DWLeaves	FWRoot	FWStem	FWLeaves
-CN	HPS	1	0.62	0.44	0.27	0.14	0.14	1.44	0.31	0.57
-CN	HPS	2	0.60	0.46	0.30	0.14	0.14	1.47	0.35	0.52
-CN	HPS	3	0.60	0.44	0.30	0.14	0.14	1.10	0.40	0.51
-CN	White	1	8.80	0.46	0.97	0.23	0.64	3.71	0.46	1.74
-CN	White	2	10.10	0.46	0.93	0.22	0.63	3.83	0.43	1.80
-CN	White	3	8.40	0.49	0.95	0.21	0.62	4.06	0.47	1.78
-CN	Red	1	11.80	0.48	0.54	0.20	0.55	1.95	0.43	1.97
-CN	Red	2	11.30	0.48	0.55	0.20	0.57	1.97	0.44	1.94
-CN	Red	3	12.30	0.47	0.55	0.21	0.57	2.05	0.45	1.96
+CN	HPS	1	2.40	0.43	0.58	0.18	0.51	1.90	0.43	1.55
+CN	HPS	2	2.20	0.43	0.58	0.18	0.46	1.94	0.45	1.39
+CN	HPS	3	2.06	0.44	0.63	0.18	0.46	1.96	0.46	1.39
+CN	White	1	8.90	0.50	1.27	0.39	0.74	4.06	0.74	1.86
+CN	White	2	8.90	0.51	1.42	0.36	0.70	4.41	0.71	1.86
+CN	White	3	9.20	0.52	1.46	0.34	0.75	4.40	0.71	1.91
+CN	Red	1	19.20	0.48	0.82	0.46	0.82	2.81	0.96	2.68
+CN	Red	2	20.60	0.50	0.79	0.44	0.82	3.14	0.94	2.47
+CN	Red	3	19.20	0.50	0.76	0.49	0.81	3.19	1.05	2.53

CN	Light	Replicate	CLeaves	CStem	CRoot	StemLphe	StemPro	StemChl	StemEleB	StemEleE	StemIso	RootLphe	RootPro	RootChl	RootEleB	RootEleE	RootIso
-CN	HPS	1	28.21	26.22	23.21	70.44	263.11	10.58	749.45	573.72	79.98	76.65	113.03	8.23	208.14	255.58	16.41
-CN	HPS	2	30.49	25.98	23.09	69.97	262.09	10.60	753.03	530.15	80.31	75.99	112.66	8.21	209.01	254.92	16.26
-CN	HPS	3	29.61	25.57	23.54	71.05	262.06	10.60	748.96	567.91	79.00	76.05	113.13	8.23	213.16	255.34	16.40
-CN	White	1	30.61	27.65	21.35	52.25	303.70	9.80	575.24	585.19	73.20	65.80	87.58	10.73	171.20	244.69	22.23
-CN	White	2	30.23	26.49	21.28	52.32	303.98	9.78	575.41	584.71	73.27	65.75	88.71	10.73	171.29	242.84	22.27
-CN	White	3	29.71	28.25	21.24	52.22	304.95	9.79	575.17	585.36	73.15	65.69	87.45	10.73	171.27	241.75	22.78
-CN	Red	1	29.55	26.08	23.00	58.46	343.33	10.30	756.93	657.37	51.82	50.87	73.67	9.41	149.17	163.14	18.85
-CN	Red	2	30.48	25.10	25.44	58.58	348.65	10.32	753.69	663.80	51.86	50.57	73.78	9.42	146.37	161.20	18.80
-CN	Red	3	29.43	26.36	25.28	58.62	350.91	10.23	759.88	645.70	53.16	50.83	73.79	9.41	145.80	161.75	18.86
+CN	HPS	1	31.38	24.57	22.35	70.42	263.10	10.58	754.31	573.99	79.99	76.50	112.43	8.23	207.87	255.81	16.39
+CN	HPS	2	30.26	25.28	21.45	69.13	260.86	10.60	754.35	535.30	80.31	76.54	112.80	8.21	208.99	254.75	16.25
+CN	HPS	3	28.05	24.66	21.44	71.66	261.85	10.60	744.16	567.62	78.99	76.04	112.95	8.23	212.76	258.39	16.40
+CN	White	1	28.78	24.94	22.19	57.97	317.11	9.64	729.78	646.77	62.62	69.97	89.75	11.51	173.18	226.38	24.77
+CN	White	2	29.10	24.67	19.95	57.92	318.37	9.63	727.81	642.92	62.47	70.65	89.79	11.52	176.48	225.69	24.75
+CN	White	3	29.56	25.57	21.82	57.98	317.06	9.64	729.67	648.95	62.86	69.44	89.39	11.50	172.98	226.75	24.94
+CN	Red	1	32.36	23.02	21.90	44.87	265.60	8.03	590.33	450.07	60.05	55.40	89.72	11.73	260.35	238.05	19.90
+CN	Red	2	29.29	24.39	21.77	45.43	265.62	8.06	590.55	450.20	60.12	55.04	89.46	11.73	257.00	240.41	19.96
+CN	Red	3	31.92	25.04	21.74	45.13	265.38	7.99	591.15	450.36	60.15	55.13	90.13	11.66	259.83	239.07	20.30
